# Fetal Growth Restriction at a Universal Late Third‐Trimester Scan and Relationship With Adverse Outcome: Retrospective Cohort Study

**DOI:** 10.1111/1471-0528.70207

**Published:** 2026-03-08

**Authors:** Elena D'Alberti, Chiara Granieri, Christos Ioannou, Christina Y. Aye, Michael Shea, Lawrence Impey

**Affiliations:** ^1^ Nuffield Department of Women's & Reproductive Health University of Oxford, University of Oxford Oxford UK; ^2^ Department of Maternal and Child Health and Urological Sciences Sapienza University of Rome Rome Italy; ^3^ Department of Obstetrics and Gynecology Fondazione Policlinico Universitario A. Gemelli IRCCS Rome Italy; ^4^ Women's Centre, John Radcliffe Hospital, Oxford University Hospitals NHS Foundation Trust Oxford UK

**Keywords:** adverse perinatal outcomes, fetal growth restriction, small‐for‐gestational‐age, stillbirth, third trimester, universal scan

## Abstract

**Objective:**

To examine the contribution of fetal growth restriction ultrasound phenotypes to adverse perinatal outcomes at term.

**Design:**

Retrospective population‐based cohort study.

**Setting:**

John Radcliffe Hospital, Oxford, UK where universal ultrasound at 35^+1^–36^+6^ weeks is performed.

**Population:**

Congenital abnormalities and births before the scan were excluded. Singleton foetuses were categorised as five mutually exclusive phenotypes using a hierarchical approach:

1. ISUOG fetal growth restriction (FGR), according to Delphi criteria;
2. Constitutional small‐for‐gestational‐age (SGA) (estimated fetal weight [EFW] < 10th centile);
3. Appropriate‐for‐gestational‐age (AGA) with either cerebroplacental ratio < 5th centile or umbilical artery >95th centile;
4. AGA with slowing abdominal circumference growth velocity (ACGV < 10th centile);
5. Normal AGA;

**Methods:**

Univariate logistic regression was employed using normal AGA as the reference group to estimate odds ratio with 95% confidence intervals. Group differences for continuous variables were assessed using mean differences with confidence intervals through a generalised linear model.

**Main Outcome Measures:**

Stillbirth (SB); composite adverse outcome (CAO) (1+ of Grade 2–3 encephalopathy, cooling, ventilation > 24 h, or perinatal death); severe SGA at birth; neonatal unit admission; obstetric interventions.

**Results:**

Among 45 179 pregnancies, 54 SBs (0.1%) and 253 CAOs (0.6%) occurred. Normal AGA foetuses at the 35^+1^–36^+6^ week scan accounted for 82% of all pregnancies and for 43 (79.6%) SBs and 205 (81%) with the CAO, yet only 37.3% of neonates born with severe SGA. The absolute risk of SB and CAO was similar in all groups (0.1%–0.2%).

**Conclusions:**

Term FGR and ‘normal’ babies have similar perinatal risks, presumably because of intervention. Despite a detection rate of 62.7% for severe SGA, most adverse outcomes occurred in pregnancies with a normal scan.

## 1 Introduction

Identification of foetuses at higher risk of adverse perinatal outcomes is the cornerstone of obstetric care. Established risk factors for increased perinatal morbidity and mortality include small for gestational age (SGA), when biometry falls below the 10th centile according to a population‐based reference, and fetal growth restriction (FGR), a more complex condition defined by the Delphi criteria and characterised by multiple aetiologies [1]. Significant efforts in many healthcare systems, such as the implementation of care pathways to identify FGR, or even a universal third‐trimester ultrasound, have aimed to improve the identification of affected pregnancies [2, 3, 4]. In foetuses prenatally identified as SGA or FGR, reduced rates of stillbirth have been reported [5].

Nevertheless, the association at least between SGA and perinatal death is much weaker at term than it is preterm, with only 30% of term stillbirths reported as SGA in a recent nationwide cohort study [6], with an unknown but presumed higher proportion of babies with FGR. This has driven the search for alternative ultrasonographic markers, such as cerebroplacental ratio (CPR) [7] or fetal growth velocity (FGV) [8], to identify a wider spectrum of placental dysfunction, and enable a better risk stratification of appropriate‐for‐gestational age (AGA) foetuses. It remains unclear, however, how many term stillbirths have identifiable FGR and what level of risk is associated with different classifications of FGR.

The aim of this study was to assess the contribution of antenatally diagnosed FGR to adverse outcomes at term in a ‘real life’ high income setting and examine the impact of different FGR phenotypes.

## 2 Methods

### 2.1 Study Design and Setting

**FIGURE 1:**
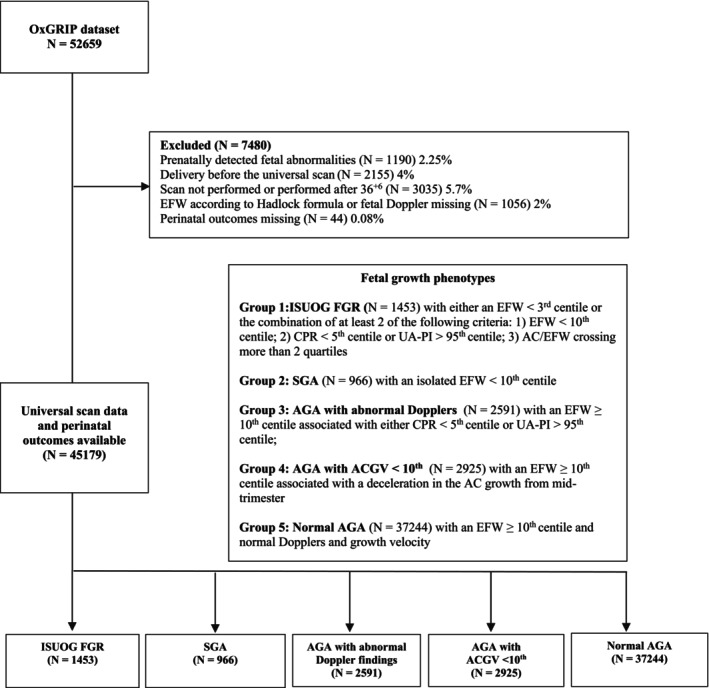
Flowchart of the study population. AC, Abdominal Circumference; ACGV, Abdominal Circumference Growth Velocity; AGA, Appropriate for Gestational Age; CPR, Cerebroplacental Ratio; EFW, Estimated Fetal Weight; FGR, Fetal Growth Restriction; ISUOG, International Society of Ultrasound in Obstetrics and Gynaecology; PI, Pulsatility Index; SGA, Small for Gestational Age; UA, Umbilical Artery.

This was a retrospective population‐based cohort study from Oxford, UK of singleton pregnancies, dated by crown‐rump length (CRL), with an estimated due date between 01/10/2016 and 31/12/2023, undergoing a ‘universal’ scan between 35^+1^ and 36^+6^ weeks. Exclusion criteria were multiple pregnancies, those with congenital abnormalities diagnosed prenatally, those who gave birth before the scan, or did not have it, and those with missing perinatal outcome data (Figure 1).

Routine antenatal care in this unit is according to UK NICE Guidelines [9] and Saving Babies Lives 3 [2]. All pregnancies also undergo uterine artery Doppler assessment at 18–21 weeks and this, together with established pre existing and new risk factors, is used to plan later ultrasound scans. In addition, all singleton pregnancies are offered a universal routine scan between 35^+1^ and 36^+6^ weeks, irrespective of prior risk. At this, the estimated fetal weight (EFW), crude growth velocity since 20 weeks, fetal Doppler velocimetry of the umbilical artery (UAPI), middle cerebral artery (MCA PI) and cerebroplacental ratio (CPR) are measured according to ISUOG Guidelines in all pregnant patients [10, 11], using quality assurance methodology [12]. Foetuses with an EFW < 10th centile [13], those with EFW ≥ 10th centile but with a fixed‐drop ≥ 40 centiles in the abdominal circumference (AC) since the mid pregnancy scan, and those with an abnormal UAPI > 95th centile [14] or abnormal CPR < 5th centile were managed according to a published protocol [15]. A low MCA PI in the absence of any other ultrasound (including low CPR) or pregnancy abnormality did not influence management. Finally, uncomplicated post‐dates pregnancies are routinely offered induction of labour or, if requested, pre labour caesarean section (CS) between 41 + 0 and 41 + 4 weeks.

Fetal growth phenotypes were categorised in groups based on established criteria for SGA and FGR patterns, according to the EFW, CPR and UAPI, and abdominal circumference growth velocity (ACGV). The ACGV was calculated as the difference in AC z‐scores between the routine 20‐week and the universal 36‐week scans. Centile thresholds of this were derived from reference charts [16], providing unconditional velocity centiles based on the absolute change in AC z‐score across this interval. Five groups were created as follows:

1. *ISUOG FGR*, defined according to the Delphi criteria, with either an EFW < 3rd centile or the combination of at least 2 of the following criteria: EFW < 10th centile [13], CPR < 5th centile or UAPI > 95th centile [14], or AC/EFW crossing more than 2 quartiles [1];
2. *SGA*, with an EFW < 10th centile, but not meeting criteria for Group 1 [1].
  The remaining AGA babies were sequentially categorised as:
3. *AGA with abnormal Dopplers*, defined as an EFW ≥ 10th centile [13] associated with either the CPR < 5th centile [14], or the UAPI > 95th centile [14]. This group was chosen as previous studies have shown these pregnancies to be at higher risk of adverse outcomes [7].
4. *AGA with abdominal circumference growth velocity (ACGV) < 10th centile*, defined by an EFW ≥ 10th centile [13] with a deceleration in the AC growth, from mid‐trimester to the routine scan, < 10th centile [16]. This group was chosen as a low ACGV has been previously shown to be a significant risk factor for composite adverse outcomes, at least among SGA foetuses [17, 18].
5. *Normal AGA*, defined as the presence of an EFW ≥ 10th centile [13] with neither an ACGV ≥ 10th centile nor abnormal Doppler indices.

To ensure mutually exclusive phenotypic categories, a hierarchical classification framework was applied to foetuses with more than one abnormal ultrasonographic feature. First, all cases fulfilling Delphi criteria for FGR were assigned to the ISUOG FGR group. The SGA group was created subsequently. Among the remaining AGA foetuses, those with abnormal Doppler indices were assigned to Group 3, including those exhibiting both abnormal Doppler findings and reduced ACGV, while foetuses with isolated ACGV < 10th centile were subsequently allocated to Group 4. The process can be seen in Tables S1 and S2.

This study was approved by the Health Research Authority (IRAS project ID: 222260; REC reference: 17/SC/0374, updated July 2024). It is reported according to the *STROBE reporting standards and* guidelines.

### 2.2 Outcomes

The primary outcomes were stillbirth and a composite of severe adverse outcomes (CAO), defined as at least one of stillbirth or neonatal death < 28 days, neonatal encephalopathy (grade 2 or 3), or therapeutic cooling or mechanical ventilation at term > 24 h. Secondary outcomes were severe SGA with a birthweight < 3rd centile [19]; and neonatal unit (NNU) admission. Process outcomes were included in the analysis to demonstrate intervention rates and included CS and induction of labour and gestational age at delivery.

### 2.3 Data Sources

Routinely collected pregnancy clinical/demographic data were extracted from Cerner Millennium (London, UK) and neonatal data were extracted from BadgerNet (Clevermed, Edinburgh, UK). Ultrasound data were extracted from ViewPoint 5 (GE Healthcare, Chicago, IL, USA).

### 2.4 Analysis

Scan findings were categorised for the mutually exclusive groups, and maternal and neonatal characteristics were summarised in each group. Continuous variables were presented as mean ± standard deviation (SD) or median with interquartile range (IQR), and categorical variables as proportions (*n* %). Missing covariate data were excluded when < 5%. Associations between fetal growth phenotypes and binary clinical outcomes were evaluated using univariate logistic regression models, with normal AGA as the reference category. Effect estimates were expressed as odds ratios (ORs) with 95% confidence intervals (CIs) using dummy coding for multi‐category comparisons. For each outcome, we additionally quantified event proportions as absolute risks for each phenotype group with 95% CI, and risk differences (RD) versus the reference phenotype, with corresponding 95% CIs. For continuous outcomes, we used generalised linear models to compare mean values across fetal growth phenotype groups. Mean differences were estimated with 95% confidence intervals using the normal AGA group as the reference. All analyses were performed using SPSS Statistics (version 30.0.0.0, Armonk, NY, IBM Corp, 2024). Statistical significance was defined as *p* < 0.05.

## 3 Results

A total of 45 179 singleton pregnancies were included. There were 54 (0.12%) stillbirths, of which 9 (16.7%) were SGA < 10th centile [19] at birth and none were SGA < 3rd centile [19]. Nine (17%) occurred at > 41 + 0 weeks and 7 were diagnosed intrapartum. Potential and certain causes of stillbirths are shown in Table S3. There were 253 (0.56%) with severe CAO; the components causing inclusion of babies with a severe CAO are shown in Table S4. Of secondary outcomes, there were 659 (1.5%) with severe SGA (birthweight < 3rd centile) [19] and 2839 (6.3%) NNU admissions. Of the total cohort, 1453 (3.2%) were classified as ISUOG FGR (Group 1) and 966 (2.1%) as constitutionally SGA (Group 2); a further 2591 (5.7%) were classified as AGA with abnormal Dopplers (Group 3), 2925 (6.5%) as AGA with ACGV < 10th centile (Group 4), and 37 244 (82.4%) classified as ‘normal’ AGA (Group 5). The relationship between the groups and overlaps is shown before and after hierarchical classification in Tables S1 and S2 respectively. The demographic and pregnancy characteristics of each phenotypic group are detailed in Table 1.

**TABLE 1:** Demographic and clinical pregnancy characteristics.

Characteristics	Group 1, ISUOG FGR (*N* = 1453)	Group 2, SGA (*N* = 966)	Group 3, AGA with abnormal Dopplers (*N* = 2591)	Group 4, AGA with ACGV < 10th (*N* = 2925)	Group 5, normal AGA (*N* = 37 244)
Maternal age (years)	30.0 ± 5.55	30.5 ± 5.38	31.35 ± 5.26	31.1 ± 5.34	31.4 ± 5.27
BMI (kg/m^2^)	24.28 ± 5.10	23.89 ± 4.57	25.71 ± 5.69	25.52 ± 5.85	25.83 ± 5.52
Smoking status	251 (17.3%)	157 (16.3%)	253 (9.8%)	326 (11.1%)	2572 (6.9%)
White Ethnicity	1020 (70.2%)	690 (71.4%)	2179 (84.1%)	2475 (84.6%)	30 841 (82.8%)
Black Ethnicity	57 (3.9%)	32 (3.3%)	49 (1.9%)	57 (1.9%)	989 (2.7%)
South‐Asian Ethnicity	197 (13.6%)	110 (11.4%)	157 (6.1%)	121 (4.1%)	1958 (5.3%)
Asian Ethnicity	71 (4.9%)	73 (7.6%)	81 (3.1%)	102 (3.5%)	1441 (3.9%)
Mixed Ethnicity	49 (3.4%)	19 (2.0%)	53 (2.0%)	76 (2.6%)	831 (2.2%)
Other or unspecified Ethnicity	11 (0.8%)	11 (1.1%)	20 (0.8%)	30 (1.0%)	411 (1.1%)
Nulliparity	787 (54.2%)	495 (51.2%)	1383 (53.4%)	1471 (50.3%)	16 566 (44.5%)
Pre‐existing diabetes	8 (0.6%)	4 (0.4%)	41 (1.6%)	12 (0.4%)	212 (0.6%)
Gestational diabetes	64 (4.4%)	38 (4.0%)	129 (5.1%)	130 (4.5%)	1915 (5.2%)
Chronic hypertension	17 (1.4%)	12 (1.4%)	44 (1.9%)	42 (1.5%)	624 (1.8%)
PIH	20 (1.6%)	14 (1.6%)	81 (3.3%)	86 (3.1%)	1006 (2.8%)
PET	181 (12.5%)	64 (6.6%)	144 (5.6%)	129 (4.4%)	1301 (3.5%)
Low PAPP‐A level	91 (9.4%)	55 (8.1%)	116 (6.3%)	109 (5.3%)	1349 (5.1%)
Unknown PAPP‐A level	486 (33.4%)	288 (29.8%)	764 (29.5%)	853 (29.2%)	10 583 (28.4%)
IMD 1–2	107 (7.7%)	62 (6.6%)	105 (4.2%)	150 (5.3%)	1759 (4.9%)

*Note:* Data are presented as mean ± SD or *n* (%).

Abbreviations: AC, Abdominal Circumference; ACGV, Abdominal Circumference Growth Velocity; AGA, Appropriate for Gestational Age; BMI, Body Mass Index; FGR, Fetal Growth Restriction; IMD, Index of Maternal Deprivation; ISUOG, International Society of Ultrasound in Obstetrics and Gynaecology; PAPP‐A, Pregnancy‐Associated Plasma Protein A; PET, Pre‐Eclampsia; PIH, Pregnancy‐Induced Hypertension; SGA, Small for Gestational Age.

Importantly, the majority of stillbirths (79.6%) and CAO (81%) occurred in the ‘normal’ AGA foetuses. Low event rates hinder comparison between FGR subtypes, subgroup estimates showing wide confidence intervals in the rate of stillbirth or the CAO between each and the ‘normal AGA’ group (Table 2). The absolute risk of stillbirth (0.08%–0.21%) and the severe CAO (0.52%–0.73%), however, was broadly similar across all groups. Overall, there was no difference in risk of stillbirth between the 4 FGR subtypes combined (0.14%) and the normal AGA babies (0.12%) (OR 1.20; 95% CI 0.62–2.33); the same applies to the CAO with absolute risks of 0.60% and 0.55% respectively (OR 1.10; 95% CI 0.80–1.51). Of the 9 (16.7%) stillbirths who were SGA at birth (< 10th centile) [19], four were in the ‘normal AGA’ (Group 5) group, and therefore ‘undetected’. A further one had been classified as AGA with ACGV < 10th centile (Group 4).

**TABLE 2:** Risk of primary outcomes according to fetal growth phenotypes.

	Stillbirth, *N* = 54 (0.1%)					Severe CAO, *N* = 253 (0.6%)				
	*n*/*N*	Absolute risk % (95% CI)	% of SB	OR (95% CI)	RD % (95% CI)	*n/N*	Absolute risk % (95% CI)	% of severe CAO	OR (95% CI)	RD % (95% CI)
Group 1, ISUOG FGR	2/1453	0.14% (0.00–0.33)	3.7%	1.19 (0.29–4.93)	+0.02% (−0.17 to +0.22)	8/1453	0.55% (0.17–0.93)	3.2%	1.00 (0.49–2.03)	0.00% (−0.39 to +0.39)
Group 2, SGA	2/966	0.21% (0.00–0.49)	3.7%	1.79 (0.43–7.42)	+0.09% (−0.20 to +0.38)	5/966	0.52% (0.07–0.97)	2.0%	0.94 (0.39–2.29)	−0.03% (−0.49 to +0.43)
Group 3, AGA abnormal Dopplers	2/2591	0.08% (0.00–0.18)	3.7%	0.67 (0.16–2.76)	−0.04% (−0.15 to +0.07)	19/2591	0.73% (0.40–1.06)	7.5%	1.33 (0.83–2.14)	+0.18% (−0.15 to +0.52)
Group 4, AGA ACGV < 10th	5/2925	0.18% (0.02–0.32)	9.3%	1.48 (0.58–3.74)	+0.06% (−0.10 to +0.21)	16/2925	0.55% (0.28–0.81)	6.3%	0.99 (0.59–1.65)	0.00% (−0.28 to +0.27)
Group 5, Normal AGA	43/37244	0.12% (0.08–0.15)	79.6%	Reference	Reference	205/37244	0.55% (0.48–0.63)	81.0%	Reference	Reference

*Note:* Data are presented as *n/N* (%), absolute risk, odds ratio (OR) and risk difference (RD) with 95% confidence interval (CI) according to the univariate logistic regression.

Abbreviations: ACGV, Abdominal Circumference Growth Velocity; AGA, Appropriate for Gestational Age; CAO, composite adverse outcomes; FGR, Fetal Growth Restriction; SB, stillbirth.

The risk of severe SGA (Table 3) at birth differed considerably between groups, with an OR of 27.1 (95% CI 22.4–32.8) with ISUOG FGR, but was also slightly increased with Groups 3 and 4 AGA FGR subtypes. Notably, the ‘normal’ AGA pregnancies contributed only 37.3% of all severely low birthweight [19], implying a detection rate of the 36 week scan, using all SGA or FGR subtypes, of 62.7%. Among neonatal unit admissions, the majority (78.2%) were again accounted for by ‘normal’ AGA babies.

**TABLE 3:** Risk of secondary outcomes according to fetal growth phenotypes.

	Severe SGA, *N* = 659 (1.5%)					NNU admission, *N* = 2839 (6.3%)				
	*n/N*	Absolute risk % (95% CI)	% of Severe SGA	OR (95% CI)	RD % (95% CI)	*n/N*	Absolute risk % (95% CI)	% of NNU	OR (95% CI)	RD % (95% CI)
Group 1, ISUOG FGR	222/1453	15.3% (13.4 to 17.3)	33.7%	27.12 (22.43 to 32.80)	+14.6% (12.7 to 16.5)	177/1453	12.2% (10.6 to 14.0)	6.2%	2.19 (1.86 to 2.58)	+6.2% (4.6 to 7.8)
Group 2, SGA	87/966	9.0% (7.2 to 11.0)	13.3%	14.88 (11.55 to 19.18)	+8.3% (6.5 to 10.1)	46/966	4.8% (3.5 to 6.3)	1.6%	0.79 (0.58 to 1.06)	−1.2% (−2.7 to +0.4)
Group 3, AGA abnormal Dopplers	55/2591	2.1% (1.6 to 2.8)	8.3%	3.26 (2.42 to 4.38)	+1.4% (0.7 to 2.1)	222/2591	8.6% (7.5 to 9.8)	7.8%	1.48 (1.28 to 1.71)	+2.6% (1.4 to 3.8)
Group 4, AGA ACGV < 10th	49/2925	1.7% (1.3 to 2.3)	7.4%	2.56 (1.88 to 3.49)	+1.0% (0.4 to 1.6)	176/2925	6.0% (5.2 to 7.0)	6.2%	1.01 (0.86 to 1.18)	0.0% (−1.1 to +1.1)
Group 5, Normal AGA	246/37244	0.7% (0.6 to 0.8)	37.3%	Reference	Reference	2218/37244	6.0% (5.7 to 6.2)	78.2%	Reference	Reference

*Note:* Data are presented as *n/N* (%), absolute risk, odds ratio (OR) and risk difference (RD) with 95% confidence interval (CI) according to the univariate logistic regression.

Abbreviations: ACGV, Abdominal Circumference Growth Velocity; AGA, Appropriate for Gestational Age; FGR, Fetal Growth Restriction; NNU, neonatal unit; severe; SGA, small‐for‐gestational age < 3rd centile [19].

Table 4 shows intervention rates and Table S5 shows gestational age at delivery. The absolute risk of expedited delivery by IOL or pre‐planned ELCS was higher in Groups 1 and 2, being 71.7% (95% CI 69.4–74.0) and 53.0% (95% CI 49.8–56.2), respectively. Although their mean gestational ages at birth were 1–2 days earlier, no differences in intervention rates were found when comparing Groups 3 and 4 with Group 5 (OR for induction or pre labour CS 1.03 (95% CI 0.98–1.10); OR for emergency CS 0.96 (95% CI 0.89–1.04)).

**TABLE 4:** Process outcomes according to fetal growth phenotypes.

	IOL or pre labour CS, *N* = 17 803 (39.9%)				Spontaneous onset of labour, *N* = 26 824 (60.1%)				EMCS, *N* = 6842 (15.1%)			
	*n/N*	Absolute risk % (95% CI)	OR (95% CI)	RD % (95% CI)	*n/N*	Absolute risk % (95% CI)	OR (95% CI)	RD % (95% CI)	*n/N*	Absolute risk % (95% CI)	OR (95% CI)	RD % (95% CI)
Group 1, ISUOG FGR	1026/1431	71.7% (69.4 to 74.0)	4.02 (3.58 to 4.52)	+33.1% (30.7 to 35.4)	405/1431	28.3% (26.0 to 30.6)	0.25 (0.22 to 0.28)	−33.1% (−35.4 to −30.7)	309/1453	21.3% (19.2 to 23.4)	1.55 (1.36 to 1.75)	+6.4% (+4.3 to 8.5)
Group 2, SGA	503/949	53.0% (49.8 to 56.2)	1.79 (1.57 to 2.04)	+14.4% (11.2 to 17.6)	446/949	47.0% (43.8 to 50.2)	0.56 (0.49 to 0.64)	−14.4% (−17.6 to −11.2)	143/966	14.8% (12.6 to 17.0)	0.99 (0.83 to 1.19)	−0.1% (−2.3 to +2.2)
Group 3, AGA, abnormal Dopplers	1060/2569	41.3% (39.4 to 43.2)	1.12 (1.03 to 1.21)	+2.7% (0.7 to 4.6)	1509/2569	58.7% (56.8 to 60.6)	0.89 (0.83 to 0.97)	−2.7% (−4.6 to −0.7)	471/2378	19.8% (18.2 to 21.4)	1.27 (1.15 to 1.41)	+4.9% (+3.3 to 6.6)
Group 4, AGA ACGV < 10th	1002/2896	34.6% (32.9 to 36.3)	0.84 (0.78 to 0.91)	−4.0% (−5.8 to 2.2)	1894/2896	65.4% (63.7 to 67.1)	1.19 (1.09 to 1.29)	+4.0% (2.2 to 5.8)	379/2925	13.0% (11.7 to 14.2)	0.85 (0.76 to 0.95)	−1.9% (−3.2 to −0.6)
Group 5, Constitutional AGA	14 212/36782	38.6% (38.1 to 39.1)	Reference	Reference	22 570/36782	61.4% (60.9 to 61.9)	Reference	Reference	5540/37244	14.9% (14.5 to 15.2)	Reference	Reference

*Note:* Data are presented as *n/N* (%), absolute risk, odds ratio (OR) and risk difference (RD) with 95% confidence interval (CI) according to the univariate logistic regression.

Abbreviations: ACGV, Abdominal Circumference Growth Velocity; AGA, Appropriate for Gestational Age; CS, C‐section; EMCS, emergency C‐section; FGR, Fetal Growth Restriction; IOL, induction of labour.

## 4 Discussion

### 4.1 Main Findings

In this study we defined five fetal growth phenotypes at a universal late pregnancy scan, and assessed their association with adverse perinatal outcomes in a population of over 45 000 singleton pregnancies. The principal findings are that (1) the risks of both stillbirth and the CAO were similar when all FGR subtypes are compared with normal AGA babies and that (2) the majority of severe clinical adverse outcomes occur in pregnancies classified at the 36 week scan as ‘normal’ AGA. That this is not primarily due to poor scan performance is implied by the high detection rate of severe SGA at this scan [20, 21], if all SGA or FGR subtypes are classified as screen positives.

### 4.2 Interpretation

These findings likely reflect both true residual risk that is not identifiable with current biometric and Doppler markers, and attenuation but not elimination of risk among the more abnormal phenotypes due to earlier intervention [22].

Comparison of the risks among the four FGR subtypes is limited because of the small numbers with wide, overlapping confidence intervals and because of differing intervention rates. Nevertheless, this intervention in the higher‐risk subgroups is probably responsible for their low rate of adverse outcomes. In the ISUOG FGR Group 1, 71.5% have their birth expedited with a mean gestational age at birth 12 days before the ‘normal’ AGA group. Their mortality is 0.14%, a figure that might be reduced further with the earlier birth that is probably appropriate in this group [1, 4]. The constitutional SGA Group 2, with birth at a gestation more in line with existing literature, has similar risks. Their persistent contribution to adverse outcomes undermines current practice to differentiate at 36 weeks between pathological and constitutional SGA [23].

In the cohort, only 16.7% of stillbirths were SGA; none had severe SGA. This is lower than usually quoted among term stillbirths [6]. It likely reflects the higher than anticipated [24] severe SGA detection rate of 67.2%. Nevertheless, undetected SGA remains a significant problem. Detection might be improved by repeating the scan in selected women, for our previous analyses showed that pregnancies with an EFW between the 10th and 20th centile at the 36 week scan have a near 10‐fold increase in the risk of severe SGA at birth (aOR 9.45; 95% CI 7.11–12.54) [25]. This is higher than the risk of severe SGA among our AGA suspected FGR groups (OR 3.26; 95% CI 2.42–4.38 and OR 2.56; 95% CI 1.88–3.49). Nevertheless, the small contribution to adverse outcomes and absence of severe SGA among stillbirths, and the relatively small proportion of even SGA < 10th centile (16.7%), all point to the limitations of size, whether ultrasound‐estimated or birthweight, as a risk factor. Indeed, although an EFW 10th–20th centile was a risk factor for severe SGA in our previous analysis [25], this risk did not extend to stillbirth (OR 1.35; 95% CI 0.53–3.41) or the more frequent composite severe adverse outcome (OR 1.05; 95% CI 0.64–1.70).

The concept of FGR as opposed to size as a risk is widely supported [1, 8]. Groups 3 and 4 were constructed (after exclusion of Group 1) in line with existing literature suggesting increased risk: those where growth may be less than intended (AGA with ACGV < 10th centile) [18] and those where Doppler abnormalities suggest other placental unit dysfunction [7]. These two ‘pathological’ AGA groups have much lower rates of intervention than the SGA babies, indeed not dissimilar to the normal AGA group (Table S1). Yet they do not have a clearly higher risk of severe adverse outcomes than the normal AGA. This partly concurs with a recent randomised trial showing planned intervention based on CPR measurement near term in non‐SGA babies did not reduce mortality, although it did reduce morbidity [26]. This and their sheer number (10% of all pregnancies), and therefore screen positives, undermines the potential of routinely using these parameters for decision making.

The burden of risk for term stillbirth and the severe adverse outcome lies with pregnancies with normal and consistent biometry, and normal umbilical artery or cerebroplacental doppler by 36 weeks; indeed with babies of a ‘normal’ size at birth. This should not undermine the utility of systematic late pregnancy ultrasound screening for FGR as its potential benefits have been widely demonstrated [20, 21, 27], but it is not enough. The issue for further term mortality reduction then is prediction of risk beyond 36 weeks in babies with normal scan findings. We have previously demonstrated the importance of pre‐eclampsia and pre‐existing diabetes but these accounted for only 10% of reported stillbirths [25]. Repeat ultrasound in a subset may detect more with severe SGA but the impact on adverse outcomes will be limited; similar strategies could be used to detect large for gestational age or accelerated growth, also known to confer some additional risk [28, 29]. Indeed, comparison of growth patterns beyond 36 weeks in babies with normal and abnormal outcomes is urgently required. Nevertheless, risk prediction at term using currently available parameters remains poor.

### 4.3 Strengths and Limitations

Although the majority of term perinatal mortality, severe neurological morbidity and severe morbidity have been reported to occur in infants apparently “appropriately grown” [30], the strength of our study is the prenatal phenotypic characterisation of the growth spectrum rather than considering the actual birthweight, which is only detected at birth. Other strengths are the large cohort size and the universal scan policy which eliminates selection bias. Intervention paradox, usually deemed a study limitation, was used as a variable to examine its influence in attenuating associations with adverse events and reflect what is achievable in a sophisticated but ‘real world’ setting.

We also acknowledge several limitations. (1) The above means the findings from this retrospective analysis should not be interpreted as reflective of the unattenuated overall role of growth restriction in adverse outcomes. (2) Intervention patterns as well as (3) small numbers of severe outcomes hinder inferences about different risk levels between the different FGR subtypes. (4) As with all such analyses, the accuracy of the scan will influence findings, but this reflects clinical practice and possibilities. (5) The charts used to classify both EFW and birthweight were not population‐based: this may have caused the lower than expected proportions of severe SGA as they may not fully reflect the anthropometric characteristics and growth distribution of our cohort. We deliberately adopted these to maximise generalisability of our findings across clinical settings, noting the influence of intervention on birthweight distribution [31]. Finally, (6) we note a relatively high proportion of unexplained stillbirths, which is likely due to the analysis being only of term stillbirths, and the screening policy for FGR.

## 5 Conclusion

In a population universally screened for FGR around 36 weeks, most adverse outcomes occur in women with a normal result. This does not imply that the scan is ineffective, for the detection rate of severe SGA is high and the risk of adverse outcomes in pregnancies with accepted ‘abnormal’ findings was similar to those without. In the absence of better risk prediction, the biggest impact at least on stillbirth will currently only be from increased expedition of birth through routine early term induction of labour or CS [32]. This is difficult to achieve [33] and may have important adverse effects on the mother and indeed on the child in the long term [34], Achieving a balance between these and the risk of stillbirth must be for parents and society to decide.

## Author Contributions

E.D'.A.: Statistical Analysis, Study Design, Writing Draft; C.G.: Statistical Analysis, Study Design, Writing Draft; C.I.: Study Design, Writing Draft; C.Y.A.: Study Design, Writing Draft; M.S.: Statistical Analysis, Study Design, Writing Draft; L.I.: Concept, Study Design, Writing Draft.

## Funding

This study was supported by the Oxford Hospitals Charity (registered charity number 1175809). The funder of this study had no role in study design, data collection, analysis or interpretation of the data, in writing the paper, or the decision to submit for publication.

## Ethics Statement

This study was approved by the Health Research Authority (IRAS project ID: 222260; REC reference: 17/SC/0374, updated July 2024).

## Conflicts of Interest

The authors declare no conflicts of interest.

## Supporting information


**Table S1:** Overlap among fetal phenotypes groups pre‐hierarchical classification.
**Table S2:** Ultrasonographic findings among groups post‐hierarchical classification.
**Table S3:** Causes of/risk factors for stillbirth.
**Table S4:** Counts for the components of the Composite Adverse Outcome (CAO).
**Table S5:** Gestational age at delivery according to different fetal phenotypes.

## Data Availability

The data that support the findings of this study are available on request from the corresponding author. The data are not publicly available due to privacy or ethical restrictions.
