# Supplementary material for: Fetal Growth Restriction at a Universal Late Third‐Trimester Scan and Relationship With Adverse Outcome: Retrospective Cohort Study
Source: BJOG. 2026 Mar 8;133(8):1592–601. doi: 10.1111/1471-0528.70207 (PMC13254011; doi:10.1111/1471-0528.70207)
Supplement: Supplementary file 1 — Table S1: Overlap among fetal phenotypes groups pre‐hierarchical classification. Table S2: Ultrasonographic findings among groups post‐hierarchical classification. Table S3: Causes of/risk factors for stillbirth. Table S4: Counts for the components of the Composite Adverse Outcome (CAO). Table S5: Gestational age at delivery according to different fetal phenotypes. [file BJO-133-1592-s001.docx]

**Supplementary Table S1: Overlap among fetal phenotypes groups pre-hierarchical classification**

| **Phenotype** | **Total (n)** | **Ultrasound characteristics** | **n** |
| --- | --- | --- | --- |
| SGA | 2185 | Isolated SGA | 966 |
|  |  | Met Delphi criteria for FGR | 1219 |
| ISUOG FGR | 1453 | SGA | 1219 |
|  |  | AGA with abnormal Dopplers | 117 |
|  |  | AGA with ACGV <10th | 170 |
|  |  | AGA with abnormal Dopplers and ACGV <10th | 68 |
| AGA with abnormal Doppler | 2708 | Isolated abnormal Dopplers | 2378 |
|  |  | Met Delphi criteria for FGR | 117 |
|  |  | Associated ACGV <10th | 213 |
| AGA with ACGV<10^th^ | 3308 | Isolated ACGV <10th centile | 2925 |
|  |  | Met Delphi criteria for FGR | 170 |
|  |  | Abnormal Dopplers | 213 |

**ACGV:** Abdominal Circumference Growth Velocity; **AGA:** Appropriate for Gestational Age; **FGR:** Fetal Growth Restriction; **ISUOG:** International Society of Ultrasound in Obstetrics and Gynecology; **SGA:** Small for Gestational Age; *of which 2420 (93.4%) had abnormal CPR and 171 abnormal UA-PI;

**Supplementary Table S2: Ultrasonographic findings among groups post-hierarchical classification**

| ***Characteristics*** | **Group 1**  **ISUOG FGR**  **(N =1453)** | **Group 2**  **SGA**  **(N = 966)** | **Group 3**  **AGA with abnormal Dopplers**  **(N = 2591)** | **Group 4**  **AGA with ACGV <10^th^**  **(N = 2925)** | **Group 5**  **Normal AGA**  **(N = 37244)** |
| --- | --- | --- | --- | --- | --- |
| EFW < 10th centile | 1219 (83.9%) | 966 (100%) | 0 (0%) | 0 (0%) | 0 (0%) |
| EFW crossing 2 quartiles | 223 (15.3%) | 0 (0%) | 0 (0%) | 188 (6.4%) | 0 (0%) |
| AC crossing 2 quartiles* | 332 (22.8%) | 0 (0%) | 0 (0%) | 157 (5.3%) | 0 (0%) |
| ACGV < 10^th^ centile | 823 (56.6%) | 146 (15.1%) | 213 (8.2%) | 2925 (100%) | 0 (0%) |
| CPR < 5^th^ centile | 470 (32.3%) | 0 (0%) | 2420 (93.4%) | 0 (0%) | 0 (0%) |
| UAPI > 95^th^ centile** | 66 (4.5%) | 0 (0%) | 171 (6.6%) | 0 (0%) | 0 (0%) |

**Data are presented as N (%). AC: abdominal Circumference; ACGV:** Abdominal Circumference Growth Velocity; **AGA:** Appropriate for Gestational Age; CPR: cerebroplacental ratio; EFW: Estimated Fetal Weight; **FGR:** Fetal Growth Restriction; **ISUOG:** International Society of Ultrasound in Obstetrics and Gynecology; **SGA:** Small for Gestational Age; *when EFW is not crossing 2 quartiles ; **when CPR is not below the 5^th^ centile

**Supplementary Table S3: Causes of/ risk factors for stillbirth**

| **Contributing factor for stillbirth*** | **n*** | **%*** |
| --- | --- | --- |
| Pre-existing diabetes | 1 | 2% |
| Pre-eclampsia | 7 | 13% |
| AGA with possible FGR** | 3 | 6% |
| BW < 10th centile | 9 | 17% |
| BW < 3rd centile | 1 | 2% |
| Vasa praevia | 1 | 2% |
| Placental abruption | 6 | 11% |
| Cord accident | 1 | 2% |
| Chorioamnionitis | 8 | 15% |
| Fetomaternal hemorrhage | 1 | 2% |
| Intrapartum hypoxia | 4 | 7% |
| Unexplained stillbirth | 25 | 46% |

 *Not mutually exclusive categories, hence total >54

**AGA with possible FGR includes UmbArtPI>95th or CPR < 5th centile

**Supplementary Table S4: Counts for the components of the Composite Adverse Outcome (CAO)**

| **Reason for severe CAO** | **n*** | **%*** |
| --- | --- | --- |
| HIE grade 2 or 3 | 42 | 17% |
| Neonatal seizures (any type) | 71 | 28% |
| Therapeutic cooling | 77 | 30% |
| Ventilated > 24h at term | 120 | 47% |
| Stillbirth | 54 | 21% |
| Neonatal death | 25 | 10% |

*Not mutually exclusive categories, hence total >253

**Supplementary Table S5: Gestational age at delivery according to different fetal phenotypes**

|  | **Gestational age at delivery**  **(days)** | | |
| --- | --- | --- | --- |
| **Fetal Growth**  **Phenotype** | **M ± SD** | **Mean Difference** | **95% CI** |
| **Group 1**  **ISUOG FGR** | 268 ± 10 | −11.96 | (-12.42 − -11.50) |
| **Group 2**  **SGA** | 275 ± 9 | −4.73 | (-5.29 − -4.17) |
| **Group 3**  **AGA**  **abnormal Dopplers** | 277 ± 10 | −2.41 | (-2.76 − -2.06) |
| **Group 4**  **AGA ACGV <10th** | 278 ± 9 | −1.61 | (-1.94 − -1.28) |
| **Group 5**  **Normal AGA** | 280 ± 8 | Reference | Reference |
